# Ethics in Community-Based Research with Vulnerable Children: Perspectives from Rwanda

**DOI:** 10.1371/journal.pone.0157042

**Published:** 2016-06-28

**Authors:** Theresa Betancourt, Mary C. Smith Fawzi, Anne Stevenson, Fredrick Kanyanganzi, Catherine Kirk, Lauren Ng, Christina Mushashi, Justin I. Bizimana, William Beardslee, Giuseppe Raviola, Stephanie Smith, Yvonne Kayiteshonga, Agnes Binagwaho

**Affiliations:** 1 Harvard T.H. Chan School of Public Health, Department of Global Health and Population, Boston, Massachusetts, United States of America; 2 Harvard Medical School, Department of Global Health and Social Medicine, Boston, Massachusetts, United States of America; 3 Partners In Health-Rwanda/ Inshuti Mu Buzima (PIH-IMB), Kigali, Rwanda; 4 Ministry of Health, Government of Rwanda, Kigali, Rwanda; 5 Boston Children’s Hospital, Boston, Massachusetts, United States of America; 6 Partners In Health, Boston, Massachusetts, United States of America; 7 Dartmouth College, Hanover, New Hampshire, United States of America; 8 University of Global Health Equity, Kigali, Rwanda; 9 Rwanda Biomedical Center, Kigali, Rwanda; University of Kwazulu-Natal, SOUTH AFRICA

## Abstract

A “risk of harm” protocol to identify youth in need of immediate emergency assistance in a study on mental health and HIV in Rwanda among 680 youth ages 10–17 is described. Cases are presented that describe the experience in using this protocol to ensure safety of participants, with ethical and logistical challenges considered. Among the population of the study, 3.2% were deemed “risk of harm.” The most prevalent presenting problem was non-fatal suicidal behavior (91% of risk of harm cases), with 36% having a history of a reported previous attempt. Challenges included: acute food insecurity/significant poverty; lack of support/adequate supervision from family members; family violence; alcohol abuse; and HIV-related stigma. Development of a “risk of harm” protocol and collaboration between study staff, community leadership, health authorities, and health workers are critical to ensuring participants’ safety in research among vulnerable populations.

## Introduction

Rwanda is making great strides forward in its development agenda, but continues to contend with the impact of a genocide that occurred in 1994 which has been exacerbated by the impact of HIV/AIDS on certain segments of the population. In the context of such compounded adversity, the implications for the mental health needs of children, adolescents, and families are significant [1–3]. Given limited empirical information on the mental health of children and adolescents in Rwanda, research is needed to identify the distribution and determinants of mental health problems in order to inform service delivery and strengthen systems of care. Research can also illuminate needs for mental health services for children and adolescents which can be of value in policy and program design [4].

Mental health care is integrated in all national health system structures and at the community level. Several clinical education programs for mental health and general professionals have been implemented, and dedicated referral facilities have been established. Decentralized hospitals and health centers across the country are now capacitated to provide mental health care. Progressive national policy goals include decentralizing health services to the district level, instituting a community-based insurance scheme, integrating clinical services, coordinating donors based on Ministry of Health policy goals, focusing on performance-based financing, and achieving middle-income country status. Official procedures exist to guide the referrals of individuals requiring mental health services to the appropriate level of care, with the most specialized mental health care available at the central level [5]. Financing for the mental health care system is part of the broader health care system that is covered through a progressive national community-based health insurance scheme, *mutuelle de santé* [6,7].

The purpose of this paper is to discuss challenges and successes in carrying out mental health research among vulnerable children and adolescents in Rwanda and implementing a risk of harm (ROH) study protocol in coordination with government and local partners. Although documenting the burden of mental health problems among children and adolescents is important for advocating for services, it is to be expected that in the process of data collection, cases will emerge that reflect an acute risk of harm, as well as more chronic problems, such as limited household resources and difficulties in accessing existing services. From an ethical standpoint, and as required by both international and local ethics review boards, it is important that research studies with vulnerable groups anticipate and plan for both acute and chronic situations of risk and the ethical dilemmas that arise. If researchers plan in advance how the study will address some of the critical situations when they occur, they can in part address the challenge of what Richardson refers to potential “ancillary-care obligations” to study participants [8]. The challenge remains, however, when implementation of existing plans cannot address the acute situation; in this regard, having prior relationships with local providers is imperative to collaboratively troubleshoot how these service needs can be met.

It is helpful to frame some of the ethical challenges and dilemmas that can occur in work with vulnerable populations in low and middle-income country (LMIC) settings conceptually. Emanuel et al. (2004) [9] proposed eight specific principles that are useful for framing the ethical issues that often arise in such work. For instance, the first principle lays out the importance of collaboration with local partners including policy makers, academics, and the communities in which the research will be conducted. A second domain of the Emanuel framework pertains to ensuring the research has value to the participants–for instance working with existing systems to build their capacity. Other components include core principles of all ethical research including ensuring scientific validity, justice, beneficence, independent reviews of the protocol, and informed consent/assent of study subjects. Finally, the model underscores that research must be conducted with respect for the local communities and particularly for the study participants.

Using the Emanuel framework as a lens for analysis and building off of other writings that have adapted the framework for addressing ethical issues arising in psychosocial research [10], we will present a summary of the field conditions, ROH situations, and issues for referral that occurred over the course of an epidemiological study on children directly and indirectly affected by HIV/AIDS in Rwanda (outlined below). Cases that illustrate some of the ethical issues that have emerged in this research will be described and lessons learned from this process, as well as issues for future research will be discussed.

## Method

A matched case-control study was conducted (March 2012 –December 2012) in the Kayonza and Kirehe districts in rural Rwanda to estimate the prevalence and examine risk factors of mental health problems among children and adolescents ages 10–17 who were: 1) living with HIV; 2) HIV-affected (i.e. those who had an HIV-positive caregiver, but who had negative serostatus themselves); and 3) non HIV-affected (i.e. those who were not HIV-infected or affected as outlined above) [11]. All HIV-positive children and adolescents in the study were being followed up by our collaborating non-governmental organization, Partners in Health, for HIV related care. This list of HIV-positive youth was derived from the Kirehe and Rwinkwavu District hospitals PIH Electronic Medical Record (EMR) and was used for outreach and recruitment into the study.

A risk of harm protocol was developed for the study collaboratively between the non-governmental organization, Partners In Health—Inshuti Mu Buzima (PIH-IMB), local health care providers, and the study team to provide field staff with a structured approach to addressing high risk situations and ensuring safety and appropriate referrals to available services. This protocol was devised based on established protocols used in Rwanda and in other studies based in sub-Saharan Africa [12,13] and was adapted for use in the present study by the Rwandan research team with input from clinicians at PIH-IMB. The overall research protocol (Protocol #15440), and the specific plans for the ROH protocol were reviewed and approved by the institutional review boards (IRBs) at the Harvard T.H. Chan School of Public Health and the Rwandan National Ethics Committee (RNEC) through the National Research Committee in the Ministry of Health. Informed consent was written and was observed by a witness who was a member of the research team; for participants who were illiterate a fingerprint was accepted in lieu of a signature. Child informed assent followed the same procedures. Great care was taken to conduct the child informed assent and caregiver informed consent processes in an independent fashion to reduce risk of making children feel pressured to participate if their caregiver gave consent. To overcome issues with low literacy, the informed consent/assent form was read aloud in Kinyarwanda to all prospective participants (separately) by trained research assistants. During the consent/assent process, all participants were informed that information shared during the study was private, however, confidentiality would need to be broken if the participant or someone else might be in danger. The IRBs approved this consent procedure.

“Risk of harm” in this case was defined as conditions that involved potential “life or death” situations related to probable harm to the physical integrity of study participants, usually due to non-fatal suicidal behavior (NFSB), but also related to risk of physical or sexual abuse, serious harm against another person, being a victim of a serious crime, and urgent unmet medical needs. According to the protocol, individuals who were considered as being at risk of harm were referred for appropriate mental health and health care and the team worked closely with the family and community leaders (as appropriate) as well as actors within the health system to ensure safety, security, and access to other needed services as deemed appropriate.

In most instances, cases were identified by research staff using the ROH protocol which involved further probing if a participant endorsed items asking about moderate or high NFSB on two depression measures (an adapted version of the Center for Epidemiological Studies Depression Scale for Children [14] and an adapted version of the internalizing scale of the Youth Self-Report [15] or via information shared during the process of administering the survey measures. A decision tree flowchart of this ROH protocol, as shown by Betancourt et al. [16] was used in this study to further assess and respond to child and adolescent vulnerability due to immediate risk of harm. Children who were identified as at potential risk of harm were further screened by study staff and categorized according to the protocol as requiring referral or at low/no risk per the protocol.

## Results

Of the 680 children in the final sample, 22 (3.2%) were deemed “risk of harm” (see Table 1). Over 63% of the ROH cases were female with a mean age of 15 (range 11–17). The primary reason for risk of harm classification was non-fatal suicide behavior n = 20 (91%). Thirty-seven percent (n = 8) of the children reported having previous suicidal attempts, with drowning in the river being the most commonly reported method of attempting suicide (n = 4).

**Table 1:** Summary of Risk of Harm (ROH) Cases.

Out of 680 Children, 22 were initially considered Risk of Harm Cases = 3.2%.		
	N	%
**Demographics**		
Female	14	63.6
Age (Range = 11–17, mean = 15.05)		
HIV Infected	10	45.5
HIV Affected	9	40.9
Not Affected	3	13.6
**Reason for ROH classification** (children can have multiple reasons)		
Non-Fatal Suicidal Behavior (NFSB)	20	90.9
Physical Abuse	4	18.2
Confusion over HIV status	4	18.2
Caregiver Domestic Violence	3	13.6
**Referrals for ROH Cases** (children can have multiple referrals)		
Hospital Mental Health/Social Work Team	11	50.0
Health Center	10	45.5
Community Health Worker (CHW)	9	40.9
Village Leader	3	13.6
*Accompagnateur* (specialized CHW for HIV)	2	9.1
Police	2	9.1
No Referrals (deemed low/no risk following assessment)	2	9.1
**Reported Previous Suicide Attempt**	8	36.4
Drowning	4	18.2
Mouse Poison	1	4.5
Burning with Boiling Water	1	4.5
Unknown	2	9.1
**Identified Reason for NFSB (out of 20 cases reporting current suicidality)** (children can have more than one)		
Parental Absence	14	70.0
Death	6	30.0
Abandonment	5	25.0
Reason Unknown	3	15.0
Physical Abuse	10	50.0
HIV-related Stigma	9	45.0
Caregiver Domestic Violence	5	25.0
Alcohol Abuse in Family	4	20.0
Food Insecurity	4	20.0
Lack of School Fees	3	15.0
Reintegration of Parent after Incarceration	3	15.0
Suicide/Attempt in Family	2	10.0
Housing Insecurity	1	5.0

According to the youth, reasons for NFSB were parental absence resulting from death, abandonment, or unknown reasons (n = 14, 70% of the children with ideation), physical abuse (n = 10, 50%), and HIV-related stigma (n = 9, 45%). Domestic violence and alcohol abuse in the family were also identified as reasons for NFSB (25% and 20% respectively). Poverty, including food insecurity, and challenges with housing or lack of school fees, were also cited as causes of NFSB (n = 6, 30%). Two children that were HIV-positive were classified as ROH after they claimed that they were HIV-negative and in both instances had stopped taking prescribed antiretroviral medication.

### Case Studies

The present set of case studies focuses on the range of child and adolescent ROH cases addressed during the study. Case studies are typically used when examining a topic in-depth or at a level of complexity that is not feasible through quantitative research [17]. This method allows for a deeper analysis of specific areas of inquiry. In this regard, the goal is to provide detailed descriptions of ROH cases among youth in order to shed light on possible strategies to improve health, safety, and quality of life in this vulnerable group. Each case touches upon issues involved in making referrals for further services in this context and how the study team worked within the health care system to overcome some of the difficulties and ensure the safety of the young person participating in the study.

Janine (pseudonyms are used throughout to protect participant confidentiality) was a 16 year old girl who was enrolled in the study given her family’s history of HIV. Upon completing the questionnaire, it became evident to the survey team that Janine’s suicidal ideation was active and serious. She admitted to having made several previous suicide attempts by attempting to drown herself in the river. Her mother was physically and verbally abusive towards Janine. The mother also continued to have serious conflicts with Janine’s father, who had left the home.

Janine’s case was challenging since the field staff encountered her towards the end of the day as many local clinics/health services were closing. The team made contact with mental health services at the District Hospital that agreed to refer Janine early the following morning. Given that her parents could not ensure her safety, the research team reached out to the head community health worker (CHW) responsible for the village to ensure that no harm came to the girl in the interim. The CHW monitored Janine closely overnight until she could be taken to the hospital for further assessment and care. Field staff worked with the CHW and the girl to access health insurance, so that she was able to obtain prescription drugs and receive mental health care and social work assistance at the hospital. Janine was then able to receive follow-up and ongoing mental health care from a hospital specialist.

Janine’s case represents how the system was able to respond in supporting and stabilizing a vulnerable young person even in the absence of a supportive family. In this instance, as in many such cases when family functioning was poor, the Rwandan system of community health workers became a critical point of ensuring safety and stability. Such approaches are consistent with the framework presented by Emanuel et al. (2004) [9] as well as by Wassenaar & Mamotte (2012) [10].

In addition to the immediate risk of harm, significant poverty was reported in some cases, as reflected in Jonas’ situation, which was related to alcoholism in the family. For Jonas, significant housing and food insecurity were pervasive problems leading to his thoughts of ending his life:

Jonas was a 13 year old boy who reported that he would sometimes spend several days without eating since he did not have enough food. The family had problems in accessing health care since they did not have health insurance. The social worker at the hospital had attempted to enroll the family in free health insurance, however the parents failed to follow through with all the steps for registration. Although the father was employed, the family’s poverty was exacerbated by the father spending the family’s income on alcohol. Jonas was the older of two children in the home.

When the field staff met Jonas, he shared that he had already attempted suicide several times. He said that uncertainty regarding housing and food insecurity were the reasons he wanted to die. The field staff contacted the mental health department at the Rwinkwavu District Hospital to make an urgent referral. Follow-up with field staff indicated that the boy was not actively suicidal at the time of assessment. Although he had thought of suicide a week prior, he said he did not go through with it because he felt responsible for taking care of his younger sibling. Field staff facilitated the referral to the mental health department at the district hospital, setting up an appointment for Jonas and then calling his CHW to bring him to the mental health team the following morning. The CHW brought Jonas for an initial appointment there, with a follow-up appointment scheduled two weeks later.

Jonas’ situation was similar to other ROH cases in this study in that alcoholism compounded the issues of violence and poverty within the family and reflected the complex range of challenges that families faced. In some cases, reduced support from family members was compounded by illness. For example, HIV infection also played a role in the ROH cases encountered:

Jean was a 15 year old boy living with HIV who was orphaned by both parents when he was only two months old. He mentioned that he had thoughts of suicide related to stigma from his neighbors who were excluding him from eating with their children because he was HIV infected. He also experienced HIV-related stigma at school, which resulted in him dropping out. He would often mention to his grandmother, whom he was living with, that he was going to commit suicide. Jean was also not adhering to his HIV regimen; although his suicidal ideation was not acute, he was at significant risk of harm through his non-adherence to antiretroviral therapy.

Field research staff worked closely with the local health center and referred him to the social worker there. The nurse and the social worker at this health center were provided with a detailed background of his case by research staff and he was later transferred to the mental health department at the district hospital by the health center staff. In addition, research staff engaged a local community health worker, who agreed to watch over him and follow-up with his *accompagnateur* (CHW trained in HIV services) to address his non-adherence to HIV treatment. (The *accompagnateur* is the backbone of the PIH-IMB community-based health care model. S/he is trained to recognize the symptoms of illness or side effects of medication. Maintaining patient confidentiality, s/he provides emotional support to patients and works to improve adherence to treatment. S/he is the link between the clinician and the community, and maintains contact with patients.)

The isolation related to HIV stigma in Jean’s case appeared to contribute to his non-adherence to treatment and his suicidal ideation. However, the field staff was able to liaise with local health care workers to effectively refer him for care in the health care system. Engaging the local CHWs was also an important step to ensure that issues of community relationships and stigma in the school and community were addressed as a part of the plan to support Jean going forward. Collaboration of the research and clinical staff played a critical role in ensuring access to care for an immediate risk of harm case identified by the study.

In addition to HIV-stigma experienced at school and in her community, another youth’s experience was compounded by being subjected to physical abuse at home.

Clementine, a 17 year old girl, reported attempting to commit suicide several months prior to her interview by trying to drown herself in the Akagera River. Other children treated her poorly and shunned her because she was living with HIV. In a recent incident when children were harassing her, Clementine became angry and began fighting, resulting in one of the children being injured. Clementine’s father said that he would beat Clementine for the incident when they got home. Fearing her father’s harsh punishment, she decided to drown herself. Her father went to look for her while she was on her way to the river and brought her home safely.

Soon after her attempted suicide, Clementine reached out to a health center social worker for help, but there were no changes in the physical and emotional abuse she experienced at home and in her community. The field team spoke to Clementine’s father, with her permission, who was surprised by the feelings she expressed and denied any physical abuse, but was open to her getting help. The field team visited the health center to discuss the case with the social worker, who was familiar with the family. In turn, the social worker made a home visit to address the family issues and physical violence in the home with particular attention to engaging the father. In addition, Clementine was referred from the health center to Kirehe District Hospital for mental health care.

## Discussion

In conducting research with vulnerable children, adolescents, and families, it is imperative that researchers anticipate urgent issues and prepare adequate protocols to respond ethically to cases where risk of harm may occur. As laid out in the framework by Emanuel et al. (2004) [9], such issues can be addressed through developing relationships with local collaborators and working with the existing system of care. Respect and care for study participants is a fundamental tenet for ensuring their well-being as a priority throughout the research.

Presented were a series of examples selected from 22 cases overall which demonstrated the range of vulnerabilities encountered in this particular epidemiological research on children directly and indirectly affected by HIV/AIDS and how the team worked within the local health care system to address them. The findings are useful for illuminating ethical concerns that may occur in other studies involving vulnerable populations in resource-limited settings. Emanuel et al. [9] argue that given the limitations in health care systems that often exist in LMICs, an ethical concern is ensuring that there is a clear plan in addressing the health care needs of study participants. This is particularly critical in the case of an immediate need for access to care.

To ensure access to care, it is necessary to carefully understand the current system and forge strong linkages in advance in order to operate successfully within it. In the examples from Rwanda, research staff members worked with the government’s health care system to lay the groundwork for the study months before it began and reached out to local CHWs, health center staff, and district level mental health teams to understand care referral processes and facilitate access to care. The project also set aside a pot of funds for transportation and other unexpected costs that were anticipated might arise in order to manage ROH cases. In addition, once ROH cases had been encountered, the study team also communicated with local providers to later follow-up on cases that had been referred. The ROH protocol facilitated closer attention to these cases.

The adaptations to the Emanuel framework which have been developed for analyzing ethical issues in psychosocial research [10] are of additional value for working in research that involves a focus on mental health. Pearson et al [18] note that an increase in monitoring and research staff competencies is necessary from an ethical standpoint for suicide-related research. Similar considerations should be made for vulnerable populations in resource-limited settings where ROH situations are likely to arise.

In addition to anticipating and addressing immediate ROH cases through referrals to the health system, researchers must also create safeguards to ensure protections for study participants living in extreme poverty, as in the case of Jonas, whose NFSB was related to severe food insecurity. This approach was consistent with the first principle of Emanuel’s ethical framework that emphasizes the importance of close collaboration with local partners and working with the existing system of care [9]. Our research team took steps to develop strong linkages to the mental health staff in the health care system to ensure that any ROH family would obtain assistance in enrolling in the national health insurance system when they were discovered to be underinsured. This is a necessary step in ensuring access to health services in Rwanda. In addition, through social work services at local health centers, we also forged linkages to food distribution and emergency feeding programs, anticipating that situations of extreme poverty and food insecurity and might also occur in the context of ROH cases.

Although research cannot directly alleviate poverty or meet unmet mental health and HIV care needs, in anticipating and understanding the available system for addressing these issues, researchers can increase the benefit/risk ratio to participants in the study, by providing appropriate referrals for primary care and basic social services. This is consistent with article 17 of the Declaration of Helsinki that describes the necessity for research to directly benefit the population when working with vulnerable communities [19,20].

There are a number of limitations of the present study. First, the case study method cannot allow one to generalize from the ROH cases presented here to other contexts more broadly. Rather the cases were selected to illuminate potential patterns in order to address or prevent similar situations within the context of Rwanda or similar research in LMIC settings in the future. Second, the sample size in both the quantitative and qualitative analysis is small (N = 22), which would indicate a low precision of the estimates in Table 1. Third, during the initial phase of the study, the research staff was learning how to work with the local health care system. Although the ROH protocol was developed to anticipate high risk cases, additional experience with these systems would have allowed for a more knowledgeable response on the part of the research team. This indicates that research studies working in similar resource-limited settings with a concomitant significant burden of high risk cases should reach out to as many local service providers as possible to learn about how to make and follow up on referrals for a range of issues. We recommend that research with vulnerable populations not begin until a basic set of relationships and a ROH protocol is in place. We also recommend setting aside flexible funds to be used to pay for transport, supplies, and even specialized yet limited health interventions as needed to ensure that the highest protections are upheld for research involving vulnerable populations. It also behooves researchers to reach out to local leaders and policy makers as feasible, to illuminate services gaps and promote a more effective response both to ROH cases. Such partnerships can help to strengthen health and social services systems through the process of research partnerships and also in dissemination of both process and outcome findings derived from work with vulnerable populations to provide evidence for action.

### Conclusion

In conclusion, there are significant challenges in carrying out research among vulnerable children and families in LMIC settings where multiple forms of adversity co-exist. In addition to the typical issues raised in study implementation, the ethical challenges outlined above may present some of the most difficult to tackle. Although there is no foolproof way of addressing all of these obstacles, researchers working among vulnerable populations should work collaboratively to ensure that ROH cases are anticipated and that a referral system is established to align with services available in the country. Building relationships between field staff and local clinical staff, CHWs, and other local leaders is a critical part of this process to prepare a clear procedure for referrals before starting a study. Through anticipating these problems, researchers can develop clear protocols to promote safety of study participants and also allocate the necessary resources, training, and supervision for field staff to implement a well thought through ROH protocol. Although these ethical challenges are difficult to navigate, they should not serve as a barrier in conducting research that can help to advocate for much needed mental health services in vulnerable populations.

## Abbreviations

ROH: Risk of Harm
RNEC: Rwanda National Ethics Committee
IRB: Institutional Review Board
PIH-IMB: Partners In Health—Inshuti Mu Buzima
NFSB: Non-Fatal Suicidal Behavior
LMIC: Low and Middle-Income Country
