# Correction: Ethics in Community-Based Research with Vulnerable Children: Perspectives from Rwanda

**DOI:** 10.1371/journal.pone.0163384

**Published:** 2016-09-15

**Authors:** Theresa S. Betancourt, Mary C Smith Fawzi, Anne Stevenson, Fredrick Kanyanganzi, Catherine Kirk, Lauren Ng, Christina Mushashi, Justin Bizimana, William Beardslee, Giuseppe Raviola, Stephanie Smith, Yvonne Kayiteshonga, Agnes Binagwaho

The first author’s name appears incorrectly in the author byline of the published article. The correct name is: Theresa S. Betancourt. The correct citation is: Betancourt TS, Smith Fawzi MC, Stevenson A, Kanyanganzi F, Kirk C, Ng L, et al. (2016) Ethics in Community-Based Research with Vulnerable Children: Perspectives from Rwanda. PLoS ONE 11(6): e0157042. doi:10.1371/journal.pone.0157042
